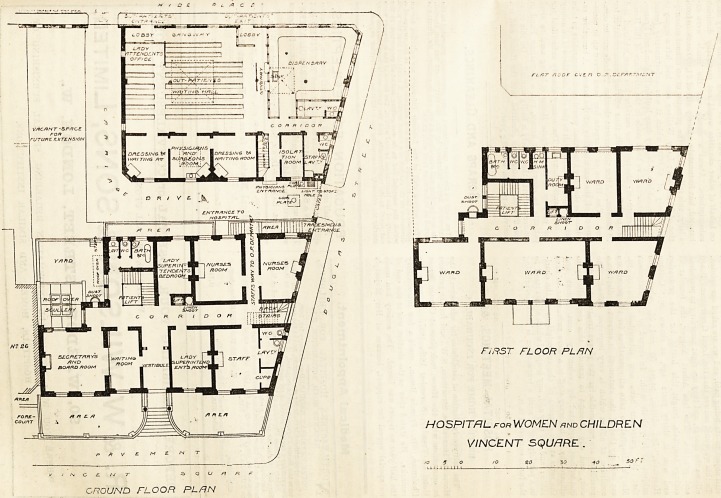# Hospital Construction

**Published:** 1897-11-20

**Authors:** 


### HOSPITAL CONSTRUCTION.

### THE GROSVENOR HOSPITAL FOR WOMEN AND CHILDREN, VINCENT SQUARE, S.W.

We referred briefly to the new buildings of this hospital in our issue of July 24th, and we now publish the plans of the two principal floors. It will be seen that the site, which has streets on three sides, is nearly covered by the buildings, although the out-patients' department, occupying nearly half the space, has only one storey above the ground. The basement, containing the kitchens, &c., is lighted by a wide area in front. The ground floor, which is approached by a flight of steps, contains in front accommodation for the administrative staff, and two rooms for the nurses and two lavatories. Some exception must be taken to one of these, which opens directly out of the room occupied by the staff, although it would appear easy to give access to it from the stairs.

At the back is the large out-patients' department referred to above, containing one surgeon's-room, two dressing-rooms, an isolation-room, and dispensary, besides a waiting-room, 42 ft. by 32 ft. There is a staff lavatory, and another presumably intended for the patients, but lighted and ventilated through the w.c. In other respects the plan is suitable and satisfactory. The two upper floors of the front building contain each five wards of varying size, with bath, closet, and lavatory accommodation, and a rather narrow duty-room; while on the second floor there is also an operating theatre. There is a patients' lift, and another for service. There is also a dust-shoot and a foul-linen shoot, appliances which had, we thought, been abandoned in modern hospital designs. The dust-shoot is external, but experience shows that it is very difficult to avoid unpleasant effects from the openings, however carefully constructed. The linen-shoot is in the centre of the building, and even if it is ventilated at the top its existence is a source of danger, and we trust its use will be abandoned. Considering the cramped nature of the site, the closets, &c., are suitably placed, though it would probably have been better to introduce the usual disconnecting lobby. The cost of the front or main building has been about £9,000, and of the out-patients' department £3,000. The architects are Messrs. Roumieu and Aitchison.

## Figures and Tables

**Figure f1:**